# Observational retrospective study on neonatal umbilical cord care: OZOILE on cord separation time and other clinical complications

**DOI:** 10.1515/med-2025-1340

**Published:** 2026-01-23

**Authors:** Gianfranco Scarpelli, Domenico Montesano, Serena Vella, Maria Lucente, Salvatore Cuzzocrea, Maria Raffaella Ramundo

**Affiliations:** Complex Operative Unit of Neonatology and Neonatal Intensive Care of the “Annunziata” Hospital – Cosenza Hospital, Cosenza, Italy; Research & Development Center, Erbagil Srl, Telese Terme, (BN), Italy; Department of Chemical, Biological, Pharmaceutical and Environmental Sciences, University of Messina, Messina, Italy; Link Campus University of Roma, Roma, Italy

**Keywords:** umbilical cord, OZOILE, stable ozonides, health care

## Abstract

**Objectives:**

Neonatal infections, which continue to be a major cause of morbidity and mortality globally, can be avoided with proper umbilical cord care. There is still no widely recognized standard for cord care despite numerous antiseptic techniques, and the hunt for safe, natural, and efficient substitutes is becoming necessary. In this regard, OZOILE, a formulation based on stable ozonides with wound-healing and antibacterial qualities, may be a viable choice. The purpose of this study was to retrospectively assess the impact of topical OZOILE on the timing of umbilical cord detachment and the avoidance or reduction of infection-related problems in newborns.

**Methods:**

The study was conducted in the Complex Operational Unit of Neonatology and Neonatal Intensive Care of the “Annunziata” Hospital-Cosenza (Italy) and included healthy newborns with a gestational age from the 35th to the 41st weeks of gestation, born to mothers with age ranging from 18 to 40 years.

**Results:**

Daily application of OZOILE spray oil, directly to the umbilical cord 3 times a day was able the stump fell off in a short time from 5 to 7 days and without any complications. OZOILE reduces secondary alterations of falling umbilical stump.

**Conclusions:**

In conclusion, OZOILE intervention could be a good candidate for umbilical cord care.

## Introduction

The umbilical cord connects the mother and fetus *in utero* and allows the exchange of nutrients and oxygen from the mother’s blood to the fetal blood, while eliminating waste materials from the fetal blood [1]. The umbilical cord is a deciduous anatomical structure, because at birth is clamped with sterile plastic forceps and then cut; what remains is referred to as the umbilical stump, undergoing, over the course of days, the anatomical-structural transformation processes essential for its fall [2]. In full-term newborns, the cord stump gradually withers and usually detaches 2 weeks after birth [3]. The term ‘cord care’ describes procedures used during the first days of life (both in hospital and at home) to ease cord separation and prevent germs from colonising the newborn’s umbilical stump [2]. Before discharge, the mother learns how to perform umbilical cord care.

Effective care of the umbilical cord prevents neonatal mortality and morbidity from bacterial infections and thus reduces the risk of neonatal sepsis and omphalitis [4]. Consequently, the application of rigorous cord-care programs became necessary at birth, during hospitalization, and on discharge. World Health Organization (WHO) recommends using a topical antimicrobial on the cord stump after cord cutting and once daily for the first 3 days [5]. However, in 2014 the Italian Drug Agency makes available new and important information aimed at minimizing the risk of serious chemical injuries associated with the use of chlorhexidine-based skin solutions in both aqueous and alcoholic solutions, for skin disinfection in newborns especially premature ones. Furthermore, antiseptic treatment has been shown to delay cord detachment due to the reduction of leukocytes required for cord separation through decreased bacterial colonization [6], 7]; this delay in stump shedding can increase postpartum care and visits, increasing the cost of postnatal care. Not only that, but the resulting delay in cord separation can also increase the risk of infections, called omphalitis, or other complications (e.g., granulomas, purulent discharge, or periumbilical erythema) [6], 8]. Omphalitis is usually caused by damp, wet conditions around the umbilical cord, which trigger the growth of bacteria and germs. Newborns are particularly susceptible to these types of infections for several reasons including an immature immune system, exposure to maternal vaginal organisms during birth, and unhygienic practices after birth [9]. Another possible complication due to delayed stump shedding is umbilical granuloma (UG), which is very common in newborns [10]. It appears as a small granulation tissue at the base of the umbilicus, consisting predominantly of fibroblasts, abundant small vessels, and endothelial and inflammatory cells. It is presumed to develop in response to a subclinical infection of the fibromuscular umbilical ring after shedding of the cord stump, leading to incomplete epithelialization of the umbilical ring and subsequent overgrowth of granulation tissue [11]. Since umbilical stump infections can rapidly progress to systemic sepsis, there is a need to find alternative strategies for optimal cord care and consequently for the prevention of neonatal sepsis.

OZOILE, stable ozonides, is a pool of lipid molecules reach in oxygen, obtained through a green technology, according to patented process by reaction of ozone with the olefin bonds of the fatty acids of the organic extra virgin olive oil +Oil^®^ obtained from native olives grown organically in Sannio (Benevento, Italy). Recently, data have been published on the isolation, identification and full structural elucidation of stable ozonides induced in superior category organic extra virgin olive oil (+Oil^®^, Erbagil Estate). In the same paper, the method to obtain pure fully characterized ozonides in the various isomeric forms is described in detail, with the advantage of using them as reference compounds for quantitative analyses. This is of fundamental importance for carrying out correlation studies between the above-mentioned stable ozonides and the related biological and health activities [12]. In literature there are studies that show the anti-inflammatory and tissue regenerating action of OZOILE also in comparison with steroids [13], 14].

OZOILE acts as powerful anti-inflammatory, reducing the levels of pro-inflammatory cytokines (TNF-α, IL 1β, IFN-g), therefore, counteracts pain, redness and burning [14], 15]. Additionally, OZOILE is an effective tissue repairer as it promotes angiogenesis increasing the transcriptional levels of VEGF and induces the correct re-epithelialization of tissues as it increases the transcriptional levels of E-cadherins, responsible for the correct adhesion between new cells [16]. Finally, OZOILE is a broad-spectrum microbicide because of the high affinity for lipoproteic components of the bacterial and fungal wall and the oxidizing action of stable Ozonides [17], 18]. Recent data also suggest that the activation of the NRF2 antioxidant pathway induced by OZOILE treatment, modifies the cellular REDOX balance by inducing a moderate oxidative stress (eustress) capable of triggering tissue regeneration processes and contributing to the regulation of inflammatory processes in foreskins affected by Lichen sclerosus (LS) [19].

The aims of this retrospective study were:

1. to observe the separation time of the umbilical stump after the application of OZOILE spray oil
2. to evaluate the reduction of local and systemic complications by the topical treatment with OZOILE

Given these considerations, the current study was created to assess OZOILE spray oil’s efficacy in encouraging prompt umbilical cord separation and averting systemic or local problems in infants. The use of stable ozonides, a novel, biocompatible substance derived from organic extra virgin olive oil that combines antimicrobial, anti-inflammatory, and tissue-regenerative properties without the disadvantages of conventional antiseptics, is what makes this research innovative. This is an alternative method for neonatal umbilical cord care that may be safer and more sustainable.

## Materials and methods

### Participants of study

This retrospective study was conducted in the Complex Operational Unit of Neonatology and Neonatal Intensive Care of the “Annunziata” Hospital -Cosenza Hospital (Italy) from 1st January 2022 to 4th of March 2024. n° 4,791 newborns with gestational age from the 35th to the 41st week of gestation, born to mothers with a chronological age ranging from 18 to 40 years and of different ethnicities.

The inclusion criteria were:

– newborns with a gestational age of 35–41 weeks;
– newborn weight of 2,400–3,800 g;
– newborns born to mothers of race ethnicity Caucasian, Romanian, Albanian, Moroccan, Ukrainian, Chinese.

The Exclusion criteria were:

– newborns with serious neonatal pathologies e.g. newborns with perinatal asphyxia, respiratory distress, metabolic disease
– newborns with any congenital disorder
– newborns of mothers with postpartum fever, mastitis, urogenital infection.

All mothers received information about the importance of umbilical cord care and the signs of its infection. Informed consent was obtained from the parents of infants.

The socio-demographic characteristics of study participants were summarized in the Table 1.

**Table 1:** Socio-demographic profile.

Age	**Minimum age**	**Maximum age**	**Mean age**			
– Maternal age	18 years	32 years	27 years			
– Gestational age	35 weeks	40 weeks	37 weeks			
Race ethnicity	**Caucasians**	**Romanians**	**Albanians**	**Moroccans**	**Ukrainians**	**Chinese**
	61.5 %	10.5 %	9.3 %	8.7 %	4.6 %	5.4 %
Type of birth	**Spontaneous**	**Cesarius**				
	76 %	24 %				
Sex of the newborn	**Males**	**Females**				
	48 %	52 %				
Birth weight	**Birth weight**	**Mean weight**				
	>2,400 kg	2,950 kg				
	<3,800 kg					
Lactation	**Breast milk**	**Artificial or formula**	**Mixed**			
	74 %	8 %	18 %			
Maternal hospitalisation						
	**Spontaneous birth without complications**	**Caesarean section**				
Time in hours	49 h	72 h				
Newborn hospitalisation time						
	**Physiological**	**Pathological**				
Time in hours	48 h	48 h				
	72 h	84 h				

### Application methods

Over the years the umbilical stump has been treated with different methods such as:

1) Dry Gauze
2) Hydrogen Peroxide
3) Denatured Alcohol
4) Sugar Salicylate, it is an impalpable powder composed of sugar and Salicylic Acid
5) Mercury Chromium (in the USA it has been abolished since 1997, because it is harmful)
6) Chlorhexidine (a note from the Italian Drug Agency AIFA *https://www.aifa.gov.it/-/nota-informativa-importante-su-medicinali-contenenti-clorexidina-11-11-2014-* ) reported a risk of serious chemical injuries when using water-based or alcohol-based chlorhexidine solutions on preterm infants);
7) No Product
8) OZOILE

Thus, in this research design, it was observed case history of subjects who were given umbilical cord care using OZOILE; subjects who were given dry umbilical cord care and subjects who were given umbilical cord care using different other treatments.

The umbilical cord was cut under sterile conditions in the delivery room. The first umbilical cord care started after the neonate had its first bath in the baby room.

In the dry method without treatments, umbilical cord was kept clean and dry and diapers were folded down under the umbilical stump so as to not irritate it and no product was applicated.

Dry gauze method consisted of leaving the umbilical cord clean and dry by covering it with a sterile gauze.

In the methods which included treatments, the products were applicated three times a day in the area of interest. In particular, OZOILE spray oil was applied directly to the umbilical cord (base and stump) 3 times a day and whenever the region is moist due to the presence of serous or bloody humoral secretions or due to the presence of organic material (urinary and fecal). It is essential to lift the clamp to allow the product to treat the entire umbilical region. Just one spray is enough, and the product is left to dry for a few minutes. It is not necessary to apply any gauze to the stump, the sanitary pad can be closed without any problems. Nurses taught the mother how to administer the assigned umbilical cord care and observe for signs and symptoms of infection at home. The cord was observed twice daily for signs of sepsis including erythema, swelling, induration and discharge, if any. The time of cord separation was noted.

The evaluation of the outcome of the topical treatment with OZOILE spray oil was carried out during the hospital stay, remembering that the physiological newborn’s stay in the ward is 49 h for the birth was spontaneous. Upon discharge for continuity of care, it was recommend using the OZOILE spray oil with the same topical application to continue until complete recovery and until the appearance of a perfect navel.

**Institutional Review Board Statement:** The study complies with the Declaration of Helsinki of 1964, revised in 2013. The parents of infants involved in this study have given their consent to participate.

**Informed consent:** The parents of infants involved in this study have given their consent to publish the data.

## Results

### Effect of OZOILE and other methods on umbilical cord fall time

**Figure 1:**
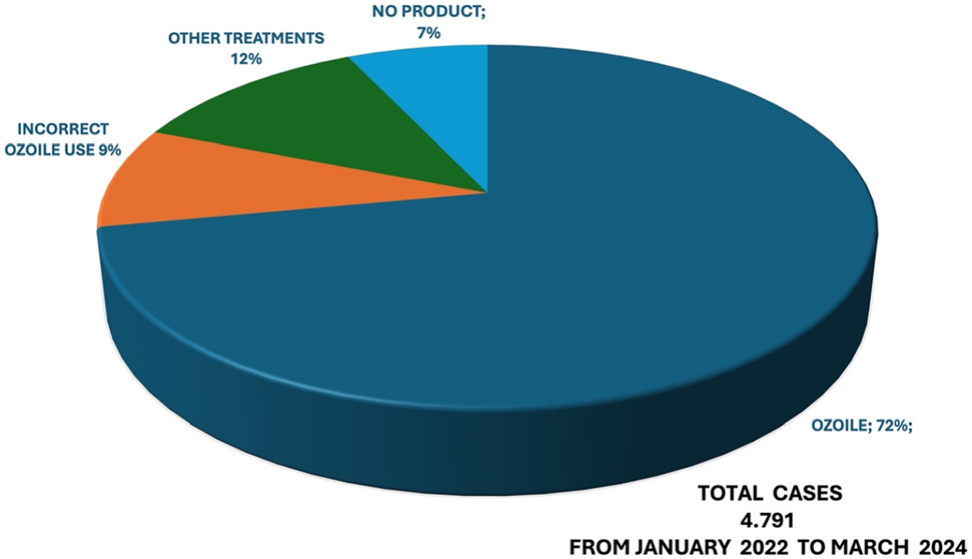
Percentages of observed cases.

The topical treatment of the umbilical stump with OZOILE began in January 2022 and ended at March 2024; from this work, a retrospective analytical study (Figure 1) was carried out in which 4,791 newborns were tested and evaluated with a percentage equal to:

1) 72 % Newborns (n. 3,450) treated exclusively with OZOILE spray oil:
2) 12 % Newborns (n. 575) treated with local treatments other than OZOILE
3) 9 % Newborns (n. 431) treated with OZOILE, but with occasional and incorrect local applications,
4) 7 % Newborns (n. 335) who were not given any treatment.

**Figure 2:**
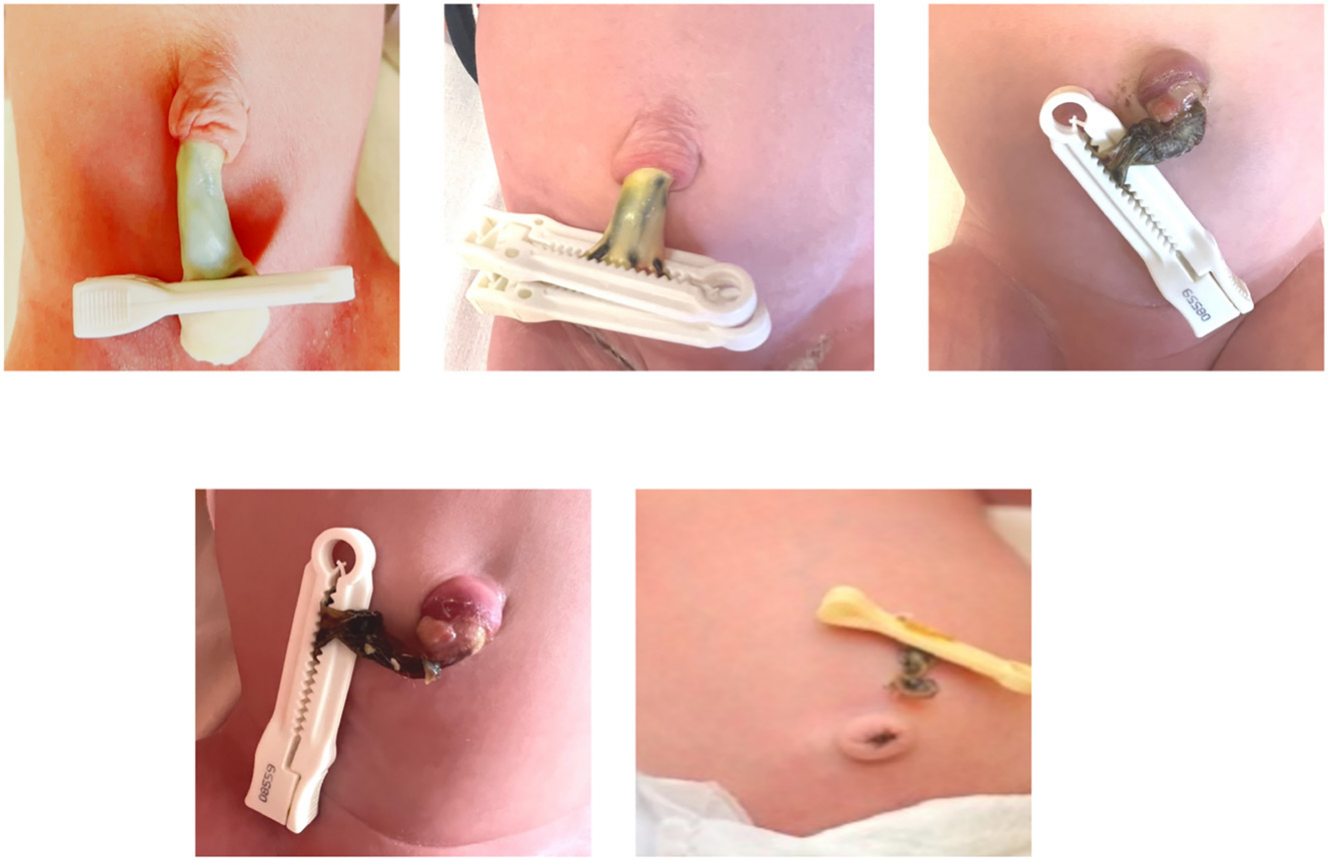
Representative images of morphological evolution of the phases of umbilical cord shedding.

After the cord is cut, the newborn is left with a stump 3–5 cm long that is treated appropriately. In the following days, the stump begins a process of drying and mummification, taking on different colors that can range from yellowish green to brown to gray/black (Figure 2).

**Figure 3:**
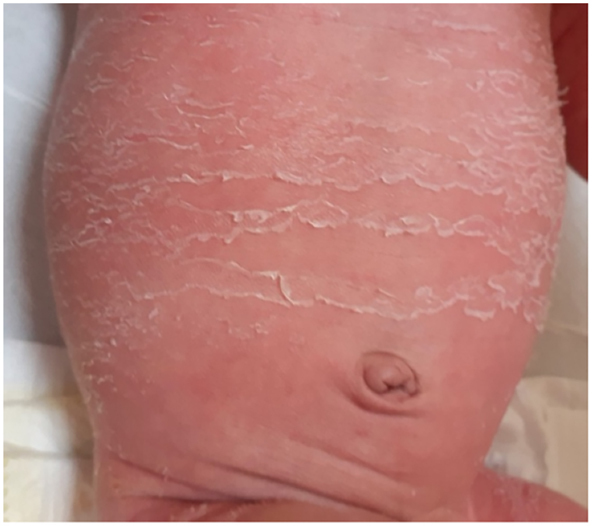
Representative image of skin alteration of the periumbilical region after treatment with other products with evident signs of dryness and flaking.

In this study, it was observed that in the 72 % of newborns treated exclusively with OZOILE spray oil, the stump fell off in a short time from 5 to 7 days and without any postnatal complications (such as cutaneous alteration, granuloma, necrotic lesions) while in the 12 % of Newborns treated with other local treatments the stump fell off after 10–12 days with the finding of local complications as well as the 7 % of Newborns who were not given any treatment, presented late times of stump fall off and onset of local inflammatory processes. In addition, it was also observed in 9 % of newborns treated with OZOILE, but with occasional and incorrect local applications, the finding of some local inflammatory manifestations (see Table 2 and Figure 3). Anyway, newborns treated with OZOILE mostly showed a shorter time for the umbilical cord to fall off compared to both those treated with other treatments and those not treated (see Table 2 and Figure 3).

**Table 2:** Table 2Different methods for umbilical cord care and adverse events.

Umbilical cord fall time	**O** **ZOILE**	**Other treatments**	**No treatment**	**Incorrect use of OZOILE**
Days	5–7	10–12	15–18	10
Adverse events	**Incorrect use of OZOILE**	**Other treatments**	**No treatment**	
– No	98 %	3 %	1 %	
– Yes	2 %	97 %	99 %	
Type of adverse events	**Incorrect use of OZOILE**	**Other treatments**	**No treatment**	
– Delayed recovery	Yes	Yes	Yes	
– Cutaneous xerosis periumbilical region	No	Yes	Yes	
– Granuloma – Necrotic lesions	No No	No Yes	Yes Yes	
Duration adverse events	**Incorrect use of OZOILE**	**Other treatments**	**No treatment**	
– Adverse event duration in days	7	10	12–15	
– Number of product application	3 times a day	3 times a day	No application	

### Effect of OZOILE topical treatment on complications relative to stump shedding

The delay in stump shedding increases the risk of infections or other complications (e.g., granulomas, purulent discharge, or periumbilical erythema). An umbilical granuloma is an overgrowth of tissue during the healing process of the belly button, that looks like a soft pink or red lump and oozing small amounts of clear or yellow fluid.

As previously mentioned, children who were treated with application methods other than topical treatment with OZOILE, showed local and systemic complications related to stump shedding. To reduce these complications, infants were additionally treated with OZOILE.

**Figure 4:**
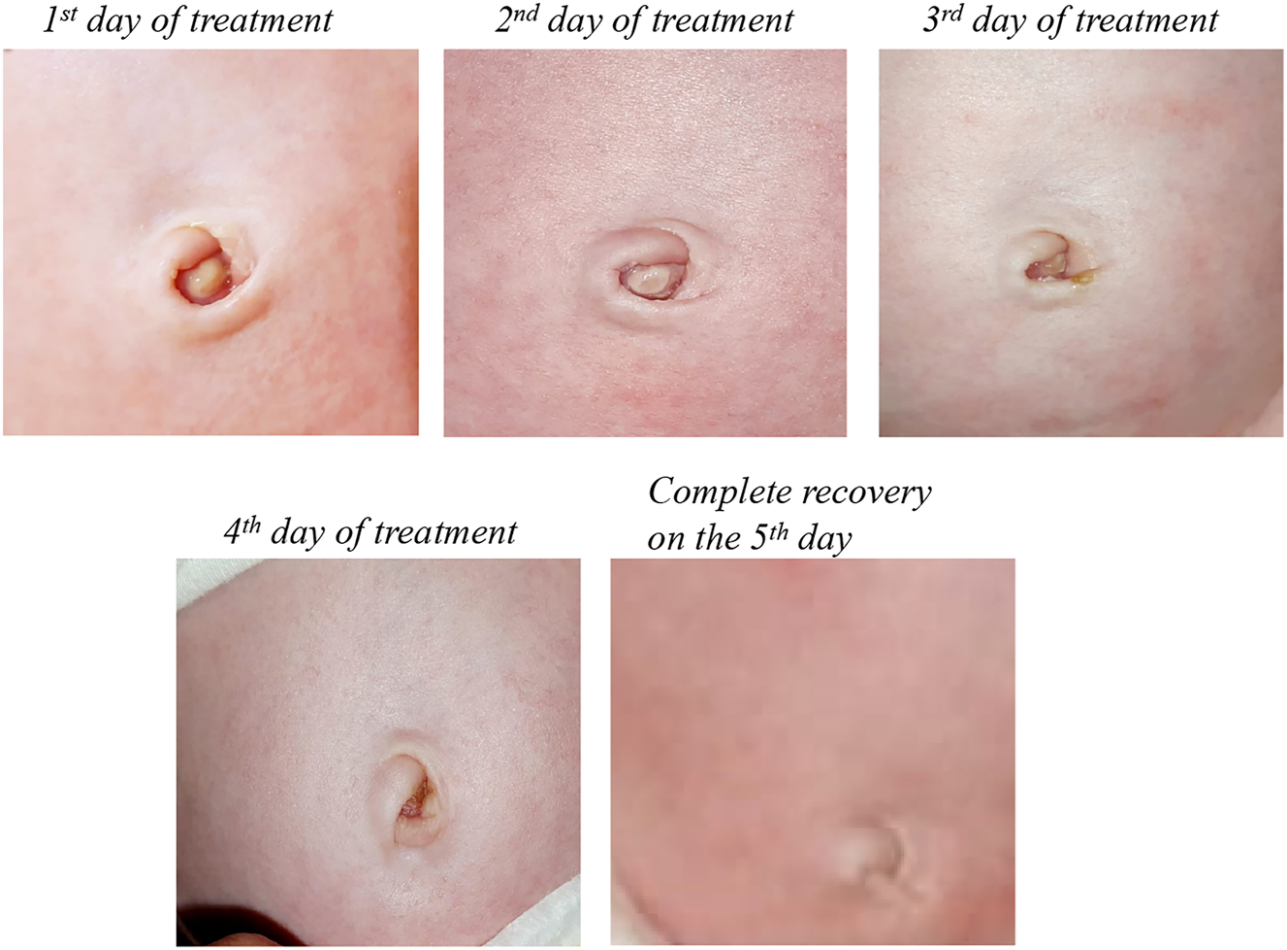
Representative images of the effect of topical application of OZOILE on umbilical granuloma.

Treatment with OZOILE, applied directly to umbilicus 3 times a day, for 5 consecutive days, was able to reduce the umbilical granuloma, inducing tissue regeneration at the umbilical level (Figure 4).

**Figure 5:**
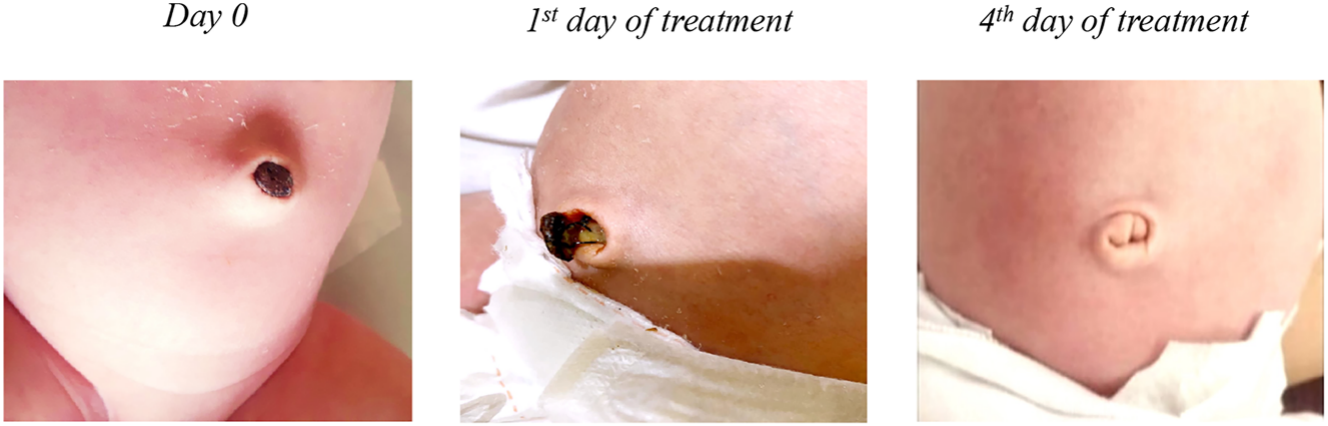
Representative images of topical application of OZOILE on necrotic lesion post-umbilical venous catheter.

The necrotic lesion post-umbilical venous catheter (UVC) is one of the most frequently used central venous access devices in the neonatal period for administering antibiotics, parenteral nutrition, and for transfusion of blood products. UVC obviates the pain, and the complications associated with repeated venous punctures. However, the placement of UVC requires experience and UVC-related complications can be very severe, including necrotic lesions. Daily application with OZOILE applied directly to umbilicus 3 times a day, reduced this complication, as showed by the fall of the necrotic lesion and regeneration of healthy tissue with a complete healing of the umbilicus at 4th of treatment (Figure 5).

## Discussion

The umbilical cord is an important bacterial colonization site, which may lead to neonatal infections such as omphalitis and sepsis [20]. A study revealed that a combined 60.2 % of all causes of neonatal deaths were due to sepsis (23.3 %) and tetanus (13 %), two prominent complications of umbilical infections [2]. Approximately 23–91 % of umbilical cords that are not treated using antiseptics will be infected by *Staphylococcus aureus* during the first 72 h after birth [21]. These bacteria can cause pustules, pyoderma, and omphalitis [22], 23]. Omphalitis is a bacterial infection of the skin and soft umbilicus tissues and surrounding regions, characterized by purulent drainage or with a stinky odor from the umbilical stump, associated with edema, erythema, and increased skin sensitivity in child’s periumbilical region, showing bleeding in some cases. The incidence of omphalitis in newborns in developed countries is 0.7 %, rising to 2.7 % in developing countries, affecting both genders equally [24]. Before the umbilical cord is separated, the remaining stump can be considered as undergoing the wound healing process and thus become a route that makes it possible for blood vessel to be infected and for the infection to be spread through the infant’s bloodstream [25]. Poor umbilical cord care causes a long delay in cord separation, usually occurring between 5 and 15 days after birth. This delay in cord separation may increase the risk of infection [6].

To date, it is still unclear as to what type of umbilical cord care is best for which babies. According to the WHO, routine cord care in developed countries usually includes daily cleaning of the cord stump with 70 % alcohol or antimicrobials such as 10 % povidone-iodine (betadine^®^), chlorhexidine, Iodine Tinstor and others which are referred to as the modern way. Meanwhile, traditional methods of cord care use honey, ghee oil, or colostrum [24], 26]. Recently, Israel and colleagues in their study showed a statistical relationship among the groups in terms of cord separation time, indicating that the mean separation times were 9.9 days (±3.3) in the povidone-iodine group, 7.7 days (±3.3) in the dry care group, and 7 days (±2.0) in the human milk group [2]. In addition, a systematic review and meta-analysis concerning application of 4 % chlorhexidine to the umbilical cord stump of newborn infants, that chlorhexidine gel use delays the cord separation time by about 2.5 d in the hospital setting [27]. Health workers in a related study reported that the application of chlorhexidine gel leaves the cord wet, and this delays the detachment of the cord and the healing process [9]. Another major problem is the ever-increasing resistance of microorganisms to the standard antibacterial preparations used in practice. In this context, the possibility of using alternative products capable of fighting the bacterial load without highlighting adverse effects, such as antibiotic resistance, would represent a preferential path that provides an optimal response to this problem. In this sense, OZOILE presents both a significant response to microorganism infections and a remedy without toxicity or allergenicity. In our study, potential benefits of OZOILE are highlighted by comparison with previously reported cord-care outcomes. In fact, our clinical observations indicate that OZOILE facilitates quick detachment without causing negative local reactions. This discrepancy might be due to stable ozonides’ different mode of action from that of traditional antiseptics. Mechanistically, the antibacterial, anti-inflammatory, and tissue-regenerative qualities of OZOILE offer a tenable biological explanation for the reported results.

The powerful antibacterial effect of OZOILE is due to the oxidation of the cell wall of microorganisms caused by the moderate oxidative stress generated by the same stable ozonides of OZOILE, as described in Saija et al. [19]. In addition to the antibacterial effects, OZOILE showed different beneficial properties such as immunomodulatory and anti-inflammatory effects, formation of new capillaries, collagen synthesis, epidermal growth and re-epithelialization [13], 14], 28]. In the literature there are several works on ozonated vegetable oils, their properties and topical applications and in particular on the use of OZOILE in the topical treatment of inflammatory states of various tissues with activation of regeneration processes and in the treatment of acute and chronic skin lesions of various etiologies [14], 15], 29]. A very recent study [12] has contributed profoundly to clarify and highlight the presence of endoperoxide structures, the stable ozonides, in OZOILE, allowing for a qualitative-quantitative analysis of these compounds, essential to closely correlate the biological and health activities, just reported, to these compounds. Together, these OZOILE effects may account for our cohort’s low incidence of secondary problems as well as quicker stump separation.

In summary, the work applied and carried out within the Complex Operative Unit of Neonatology and Neonatal Intensive Care of the Cosenza Hospital has allowed us to highlight the clinical critical issues related to the use of products other than OZOILE:

1) Falling of the umbilical stump not before 15–18 days of treatment.
2) Increase in the number of local and systemic complications
3) Presence of extensive dry and flaky skin areas in the periumbilical area.

On the contrary, the use of OZOILE has allowed us to observe:

1) Falling of the umbilical stump within 5–7 days of treatment
2) Absence of local and systemic complications

We observed that treatment with OZOILE spray oil induced a fall of the umbilical cord within 5–7 days of birth and this significantly reduces the risk of developing bacterial infections. Furthermore, we also confirmed the healing, anti-inflammatory and antibacterial effect of OZOILE, in which daily topical treatment caused a reduction or elimination of local and systemic complications. Additionally, we have not observed any side effects from the correct application of OZOILE and have not encountered similar articles in the literature with such an innovative method of umbilical care so far. Thus, because a shorter detachment time and a lower rate of complications shorten the time that a baby is vulnerable to invasive infections and may lessen the need for further postnatal consultations and therapies, these findings are therapeutically significant.

### Future perspectives

Further studies with larger, randomized populations are needed to confirm these preliminary results and to directly compare OZOILE with conventional antiseptics. The long-term effects of OZOILE on infant skin health as well as the underlying molecular pathways should be further investigated.

## Conclusions

In conclusion, the correct use of OZOILE for the treatment of the umbilical stump promotes and supports the repair and healing processes that intervene in the fall of the umbilical stump in a short time and without any complications compared to conventional treatments.

The results shown in this work suggest that OZOILE may represent an important alternative to the use of conventional antibiotic drugs, contributing to the reduction of side effects such as antibiotic resistance and drug-induced allergic phenomena in the treatment of the umbilical cord. Finally, the use of OZOILE could also significantly contribute to the reduction of health expenditure for the NHS and for families.

### Limitations study

There are some limitations to the current study. First, these data were gathered by depending on mothers’ reporting during this study because it was not always feasible to employ a direct observational method to evaluate the usage of ozoile for umbilical cord care. Naturally, parents were told to watch for any warning signs of complications and to get in touch with their healthcare provider if they had any questions before being released from the hospital. Second, because this study was not limited to moms who are actively utilizing ozoile for umbilical cord care, time can have an impact on recollection of an umbilical care routine.

Raising awareness and educating people may help the populace become more knowledgeable. Healthcare professionals are urged to offer thorough instruction since it is anticipated that as awareness of the use of ozoile in cord care grows, so will their understanding and application of it. Nurse researchers should plan studies that can shed light on how understanding ozoile use for umbilical cord care translates to its application, in addition to offering health education. It is also necessary to investigate additional parameters related to the usage of ozoile for umbilical cord care. Interpretation of these results should bring these limitations into consideration.

In addition, the products used in the current study are available on the market and were freely and independently selected by the medical staff who authored this work. The authors have no commercial relationship with the Ozoile manufacturer, and the results obtained are entirely based on their own research. No conflict of interest arose in this work.
